# Pressure support ventilation attenuates ventilator-induced protein modifications in the diaphragm

**DOI:** 10.1186/cc7010

**Published:** 2008-09-11

**Authors:** Emmanuel Futier, Jean-Michel Constantin, Lydie Combaret, Laurent Mosoni, Laurence Roszyk, Vincent Sapin, Didier Attaix, Boris Jung, Samir Jaber, Jean-Etienne Bazin

**Affiliations:** 1 General Intensive Care Unit, Hotel-Dieu Hospital, University Hospital of Clermont-Ferrand, Boulevard L. Malfreyt, Clermond-Ferrand, 63058, France; 2 Human Nutrition Research Center of Clermont-Ferrand, Nutrition and Protein Metabolism Unit, Institut National de la Recherche Agronomique, Route de Theix, Ceyrat, 63122 France; 3 Department of Biochemistry, University Hospital of Clermont-Ferrand, Boulevard L. Malfreyt, Clermont-Ferrand, 63000, France; 4 SAR B, Saint-Eloi Hospital, University Hospital of Montpellier, Avenue Augustin Fliche, Montpellier, 34000, France

## Abstract

**Introduction:**

Controlled mechanical ventilation (CMV) induces profound modifications of diaphragm protein metabolism, including muscle atrophy and severe ventilator-induced diaphragmatic dysfunction. Diaphragmatic modifications could be decreased by spontaneous breathing. We hypothesized that mechanical ventilation in pressure support ventilation (PSV), which preserves diaphragm muscle activity, would limit diaphragmatic protein catabolism.

**Methods:**

Forty-two adult Sprague-Dawley rats were included in this prospective randomized animal study. After intraperitoneal anesthesia, animals were randomly assigned to the control group or to receive 6 or 18 hours of CMV or PSV. After sacrifice and incubation with ^14^C-phenylalanine, *in vitro *proteolysis and protein synthesis were measured on the costal region of the diaphragm. We also measured myofibrillar protein carbonyl levels and the activity of 20S proteasome and tripeptidylpeptidase II.

**Results:**

Compared with control animals, diaphragmatic protein catabolism was significantly increased after 18 hours of CMV (33%, *P *= 0.0001) but not after 6 hours. CMV also decreased protein synthesis by 50% (*P *= 0.0012) after 6 hours and by 65% (*P *< 0.0001) after 18 hours of mechanical ventilation. Both 20S proteasome activity levels were increased by CMV. Compared with CMV, 6 and 18 hours of PSV showed no significant increase in proteolysis. PSV did not significantly increase protein synthesis versus controls. Both CMV and PSV increased protein carbonyl levels after 18 hours of mechanical ventilation from +63% (*P *< 0.001) and +82% (*P *< 0.0005), respectively.

**Conclusions:**

PSV is efficient at reducing mechanical ventilation-induced proteolysis and inhibition of protein synthesis without modifications in the level of oxidative injury compared with continuous mechanical ventilation. PSV could be an interesting alternative to limit ventilator-induced diaphragmatic dysfunction.

## Introduction

Controlled mechanical ventilation (CMV) has been shown to induce muscle atrophy and to alter diaphragm contractile properties [1-6], leading to early and severe ventilator-induced diaphragm dysfunction (VIDD) that has been implicated in weaning failure [7,8]. Although weaning failure may be due to numerous factors, diaphragm dysfunction induced by mechanical ventilation (MV) probably plays an important role. Indeed, animal studies reveal that 18 hours of CMV results in diaphragmatic contractile dysfunction and atrophy [9]. Moreover, the combination of 18 to 69 hours of complete diaphragmatic inactivity and MV results in marked atrophy of human diaphragm myofibers [1].

The mechanisms of VIDD have not been fully elucidated. Muscle atrophy, oxidative stress, and structural injury have been documented after CMV [7]. Muscle proteolysis is a highly regulated process accomplished by at least three different proteolytic systems: the ubiquitin-proteosome pathway, the Ca^2+^-dependent system, and the lysosomal system. All three proteolytic systems have been shown to be implicated in the increased diaphragmatic proteolysis observed after CMV, as indicated by changes in the gene expression profile of several proteolytic enzymes [10]. Muscle atrophy is not due only to an increase in proteolysis. Shanely and colleagues [11] have shown that CMV induced a rapid decreased synthesis of diaphragmatic mixed muscle protein and myosin heavy chain protein. Indeed, within the first 6 hours of MV, mixed muscle protein synthesis decreased by 30% and myosin heavy chain protein synthesis decreased by 65% [11].

MV-induced oxidative stress is also an important contributor to both MV-induced proteolysis and contractile dysfunction. Indeed, Shanely and colleagues [2] have shown that MV is associated with a rapid onset of protein oxidation in diaphragm fibers. This is significant because oxidative stress has been shown to promote disuse muscle atrophy [12] and has been directly linked to activation of the ubiquitin-proteasome system of proteolysis [13]. The precise contribution of each factor to the development of VIDD and their kinetic of apparition has yet to be defined.

Although it was demonstrated that CMV exerted several deleterious effects on the diaphragm, only few protective countermeasures have been developed to minimize CMV-induced diaphragm dysfunction and atrophy. Administration of the antioxidant Trolox has been shown to prevent CMV-induced diaphragm contractile impairments and to retard proteolysis [14]. Administration of the protease inhibitor leupeptin concomitantly with MV prevented the apparition of VIDD in rats after 24 hours of MV [15]. Intermittent spontaneous breathing during the course of CMV has been shown to protect the diaphragm against the deleterious effects of CMV [16].

In clinical practice, spontaneous breathing increases work of breathing and patients often need positive pressure ventilation to improve gas exchange [17]. The spontaneous breathing period during CMV is not always the best issue for critical care patients. In contrast, pressure support ventilation (PSV) is efficient for patients with acute respiratory failure and/or chronic obstructive pulmonary disease, even if they are anesthetized [18-20]. PSV allows diaphragmatic activity with positive pressure ventilation [21,22]. We hypothesized that PSV-associated preservation of respiratory muscle activity would induce less diaphragmatic catabolic damage as shown by modifications of proteolytic and protein synthesis activities and oxidative injury.

## Materials and methods

### Animals and experimental design

**Figure 1:**
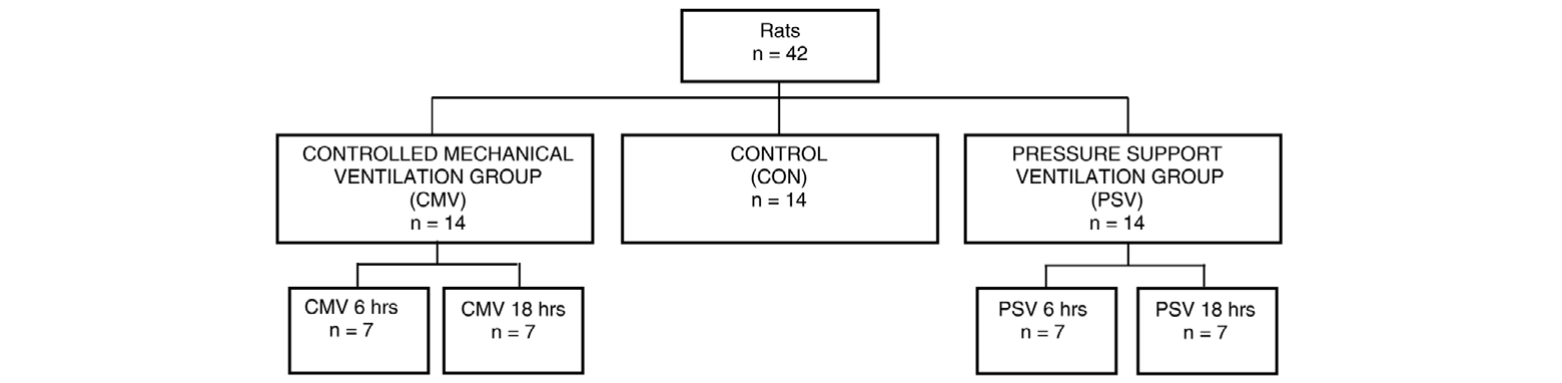
Schematic illustration of the experimental design used.

This study was performed in accordance with the recommendations of the National Research Council's *Guide for the Care and Use of Laboratory Animals *[23]. This experiment was approved by the University of Clermont-Ferrand animal use committee. Forty-two adult male Sprague-Dawley rats (250 g) were individually housed and fed rat chow and water *ad libitum *and were maintained on a 12-hour light/dark photoperiod for 1 week before initiation of these experiments. Animals were randomly assigned to 6 or 18 hours of CMV or PSV with 21% O_2 _(Figure 1). All surgical procedures were performed using aseptic techniques. After reaching a surgical plane of anesthesia (sodium pentobarbital, 50 mg/kg of body weight, intraperitoneal), animals were weighed and tracheostomized. The jugular vein was cannulated for the infusion of saline and sodium pentobarbital (5 mg/kg of body weight per hour). Body fluid homeostasis was maintained by administration of 2 mL/kg per hour intravenous electrolyte solution. The carotid artery was cannulated for measurement of arterial blood pressure, pH, and blood gas tensions (GEMpremier-3000 system; Instrumentation Laboratory, Lexington, MA, USA). Heart rate and electrical activity of the heart were monitored via a lead II electrocardiogram using needle electrodes placed subcutaneously. Throughout the ventilation period, animals received enteral nutrition (via a nasogastric tube) using the AIN-76 rodent diet with a nutrient composition of proteins, lipids, carbohydrates, and vitamins which provided an isocaloric diet (Research Diets, Inc., Brunswick, NJ, USA). Body temperature was monitored (rectal thermometer) and maintained at 37°C ± 1°C with a recirculating heating blanket. Continuing care during the experimental period included expressing the bladder, removing upper airway mucus, lubricating the eyes, rotating the animal, and passive movements of the limbs. Animals (both CMV and PSV) were regularly rotated to prevent atelectasis, to limit mechanical constraints, and to maintain ventilation/perfusion ratio homogeneity.

### Protocol for control mechanical ventilation group

Immediately after inclusion, animals were mechanically ventilated using a volume-driven ventilator (Rodent Ventilator model 683; Harvard Apparatus, Holliston, MA, USA) for 6 hours (group 1) or 18 hours (group 2). The tidal volume was 10 mL/kg of body weight and the respiratory rate was 80 breaths per minute, with a fraction of inspired oxygen (FiO_2_) of 21% but without positive end-expiratory pressure. These ventilatory conditions resulted in complete diaphragmatic inactivity and prevented noxious effects of a hypercapnia on the muscular contractile properties [2,3,24,25]. At the end of the experimental period, each animal was weighed, and the costal diaphragm was rapidly dissected and frozen in liquid nitrogen. Samples were stored at -80°C until subsequent assay (except for samples in which protein synthesis and proteolysis were analyzed, which were treated as described below). At the same time, arterial blood was obtained for culture.

### Protocol for pressure support ventilation group

Animals were also anesthetized and mechanically ventilated for 6 hours (group 4) or 18 hours (group 5) as described above (model PSV ventilator DARHD01; IFMA, Aubière, France). The level of pressure support applied, determined during preliminary studies, allowed a minute volume of 200 ± 10 mL/minute (respiratory rate of 80 ± 10 breaths per minute and FiO_2 _of 21%). The range of pressure support used was 5 to 7 cm H_2_O. The ventilator had a pressure trigger. The expiratory trigger was fixed at 25% of peak inspiratory flow, and the maximum inspiratory time was set at 1 second. The ventilator did not have back-up ventilation. If the animal was not triggering, no pressure was released. Continuing care during the experiment was also applied as above. At the end of the experimental period, the costal diaphragm was rapidly removed, dissected, and frozen in liquid nitrogen. Samples were stored at -80°C.

### Protocol for control animals

Control animals (group 3) were free of intervention before inclusion (not mechanically ventilated). These animals were anesthetized and their diaphragms were rapidly dissected, frozen, and stored at -80°C until subsequent assay. Because of the biochemical constraints (variability of the solutions of Krebs-Henselheit), each day of experimentation required a control animal.

### Tissue removal and storage

At the appropriate times (6 or 18 hours), the entire diaphragm, costal and crural, was removed, dissected, and weighed. All biochemical studies were conducted using the costal region of the diaphragm. Samples were rapidly frozen in liquid nitrogen and stored at -80°C until assay.

### Biochemical assays

#### Measurement of protein turnover *in vitro*

Proteolysis and protein synthesis were measured on the costal region of the diaphragm (approximately 250 mg). Diaphragmatic protein synthesis was evaluated by measurement of ^14^C-phenylalanine (^14^C-Phe) incorporation into diaphragm strips as described previously by Tischler and colleagues [26]. Diaphragmatic protein breakdown was measured by evaluation of the rate of tyrosine release from diaphragm samples according to the fluorimetric method of Waalkes and Udenfriend [27]. The rationale for this technique is that tyrosine is neither synthesized nor degraded by skeletal muscle and is suited as a marker of whole protein degradation [26]. Diaphragm samples were quickly removed from each experimental animal and preincubated at 37°C in Krebs-Henselheit bicarbonate buffer equilibrated with 95% O_2 _and 5% CO_2_, containing 5 mM glucose, 0.2 U/mL insulin, 0.17 mM leucine, 0.10 mM isoleucine, and 0.20 mM valine to improve protein balance [26]. After a 30-minute preincubation period, muscles were transferred to a fresh medium of similar composition but containing 0.5 mM ^14^C-Phe (Amersham Corporation, now part of GE Healthcare, Little Chalfont, Buckinghamshire, UK) to measure the rate of protein synthesis. The muscles were incubated for an additional 1-hour period. The rate of protein synthesis was determined by incubating muscles in a medium containing 0.5 mM ^14^C-Phe with a specific radioactivity in the medium of 1,500 disintegrations per minute per nanomole as described previously [28]. Tissues were homogenized in 10% trichloroacetic acid and hydrolyzed in 1 M NaOH at 37°C. Tissue protein mass was determined using the bicinchoninic acid procedure [29]. Rates of phenylalanine incorporation were converted into tyrosine equivalents, as described previously [26], and expressed as nanomoles of tyrosine incorporated per milligram of muscle per hour. Muscle protein content was measured according to the bicinchoninic acid procedure. Rates of protein breakdown were measured by following the rates of tyrosine release into the medium. At the completion of the incubation period, tyrosine concentrations were assayed by the fluorimetric method of Waalkes and Udenfriend [27]. The rates of total protein degradation were calculated by adding the rate of protein synthesis and the net rate of tyrosine release into the medium [28,30]. Rates of protein turnover were expressed in nanomoles of tyrosine per milligram of protein per hour [30].

#### Measurement of proteasome proteolytic activities

On the controlateral costal diaphragm, proteins from skeletal muscle samples were homogenized in ice-cold buffer (pH 7.5) containing 50 mM Tris, 250 mM sucrose, 10 mM ATP, 5 mM MgCl_2_, 1 mM dithiothreitol (DTT), and protease inhibitors (10 μg/mL of antipain, aprotinin, leupeptin, and pepstatin A and 20 μM PMSF [phenylmethylsulphonylfluoride]). The proteasomes were isolated by three sequential centrifugations as described previously [31-33]. The final pellet was resuspended in buffer containing 50 mM Tris (pH 7.5), 5 mM MgCl_2_, and 20% glycerol. The protein content of the proteasome preparation was determined according to Lowry and colleagues [34]. Chymotrypsin-like activity of the proteasome and the tripeptidylpeptidase II (TPPII) activity were determined by measuring the hydrolysis of the fluorogenic substrates succinyl-Leu-Leu-Val-Tyr-7-amino-4-methylcoumarin (LLVY-AMC) and Ala-Ala-Phe-AMC (AAF-AMC). To measure peptidase activity, 15 μL of the extract was added to 60 μL of medium containing 50 mM Tris (pH 8.0), 10 mM MgCl_2_, 1 mM DTT, 2 U apyrase, and 300 μM LLVY-AMC or 300 μM AAF-AMC. The activities were determined by measuring the accumulation of the fluorogenic cleavage product (methylcoumaryl-AMC) using a luminescence spectrometer FLX 800 (BioTek Instruments, Inc., Winooski, VT, USA). Fluorescence was measured continuously during 45 minutes at a 380-nm excitation wavelength and a 440-nm emission wavelength. The difference between arbitrary fluorescence units recorded with or without 40 μM of the proteasome inhibitor MG132 (Affiniti Research Projects Limited, Exeter, Devon, UK) or 100 μM of the TPPII inhibitor AAF-chloromethylketone (Sigma-Aldrich, St. Louis, MO, USA) in the reaction medium was calculated, and the final data were corrected by the amount of protein in the reaction. The time course for the accumulation of AMC after hydrolysis of the substrate was analyzed by linear regression to calculate activities (for example, the slopes of best fit of accumulated AMC versus time). Different kinetics were performed to individually measure the chymotrypsin-like activity of the proteasome and the TPPII activity.

#### Measurement of diaphragm oxidative injury

Myofibrillar protein carbonyl content was determined according to Fagan and colleagues [35] with slight modifications. Briefly, myofibrillar proteins were purified, treated with HCl-acetone to remove interfering chromophores, and protein carbonyl content was then measured using 2,4-dinitrophenylhydrazones (DNPH). Following DNPH treatment, samples were subjected to successive washings with trichloroacetic acide (TCA) 30%, TCA 10%, and four washes with ethanol/ethylacetate (1:1). The pellet was solubilized with 6 M guanidine hydrochloride and 20 M potassium phosphate (pH 2.3) through incubation at 50°C during 30 minutes. After centrifugation (800 *g *for 10 minutes at 20°C), absorbances at 280 and 380 nm were measured on the supernatant to determine protein and carbonyl content, respectively. Protein content was calculated using a calibration curve and carbonyl content using the absorption coefficient 22,000/M-cm.

### Statistical analysis

A two-way analysis of variance (StatView^®^, version 5.0; SAS Institute Inc., Cary, NC, USA) with time (6 versus 18 hours) as one factor and modality (PSV versus CMV versus control) as the other factor was used. When appropriate, a *post hoc *protected least squares difference Fisher test was used. Values are mean ± standard deviation in the text and mean ± standard error of the mean in the tables and graphs. Statistical significance was defined *a priori *as a *P *value of less than 0.05.

## Results

### Systemic and biologic response to mechanical ventilation

The principal biologic parameters are summarized in Table 1. Blood gas/pH and cardiovascular homeostases were maintained constant in all animals during CMV and PSV. There were no significant differences in total body mass between groups and no group experienced a significant loss of body mass, indicating adequate hydration and nutrition during the experimental period (Table 2). All animals urinated and experienced intestinal transit during the experimental period. All blood cultures were negative for bacteria and none of the animals demonstrated sepsis signs.

**Table 1:** Systemic and biologic response to mechanical ventilation

Biologic parameters	Control	CMV at 6 hours	CMV at 18 hours	PSV at 6 hours	PSV at 18 hours
pH	7.38 ± 0.02	7.42 ± 0.04	7.40 ± 0.05	7.39 ± 0.02	7.43 ± 0.01
PaO_2_/FiO_2_, mm Hg	360 ± 50	380 ± 40	350 ± 30	360 ± 40	370 ± 20
PaCO_2_, mm Hg	38 ± 3	40 ± 2	38 ± 3	40 ± 5	40 ± 3
MAP, mm Hg	90 ± 10	95 ± 15	97 ± 12	100 ± 10	95 ± 15
Na^+^, mmol/L	135 ± 2	138 ± 5	135 ± 3	140 ± 5	138 ± 4
K^+^, mmol/L	4.20 ± 0.1	4.0 ± 0.3	4.10 ± 0.2	3.90 ± 0.3	4.20 ± 0.2

Fraction of inspired oxygen (FiO_2_) is 21%. CMV, controlled mechanical ventilation; MAP, mean arterial pressure; PaCO_2_, arterial partial pressure of carbon dioxide; ]PaO_2_, arterial partial pressure of oxygen; PSV, pressure support ventilation.

**Table 2:** Body weight of control, pressure support ventilation, and controlled mechanical ventilation groups

Groups	Initial body mass, g*rams*	Final body mass, g*rams*
Control	253.5 ± 5.4	-
CMV at 6 hours	252.4 ± 4.5	253.5 ± 3.5
CMV at 18 hours	260.2 ± 3.2	258.6 ± 3.5
PSV at 6 hours	255.3 ± 3.8	255.4 ± 3.5
PSV at 18 hours	255.0 ± 3.0	258.3 ± 2.6

CMV, controlled mechanical ventilation; PSV, pressure support ventilation.

### *In vitro *proteolysis

**Figure 2:**
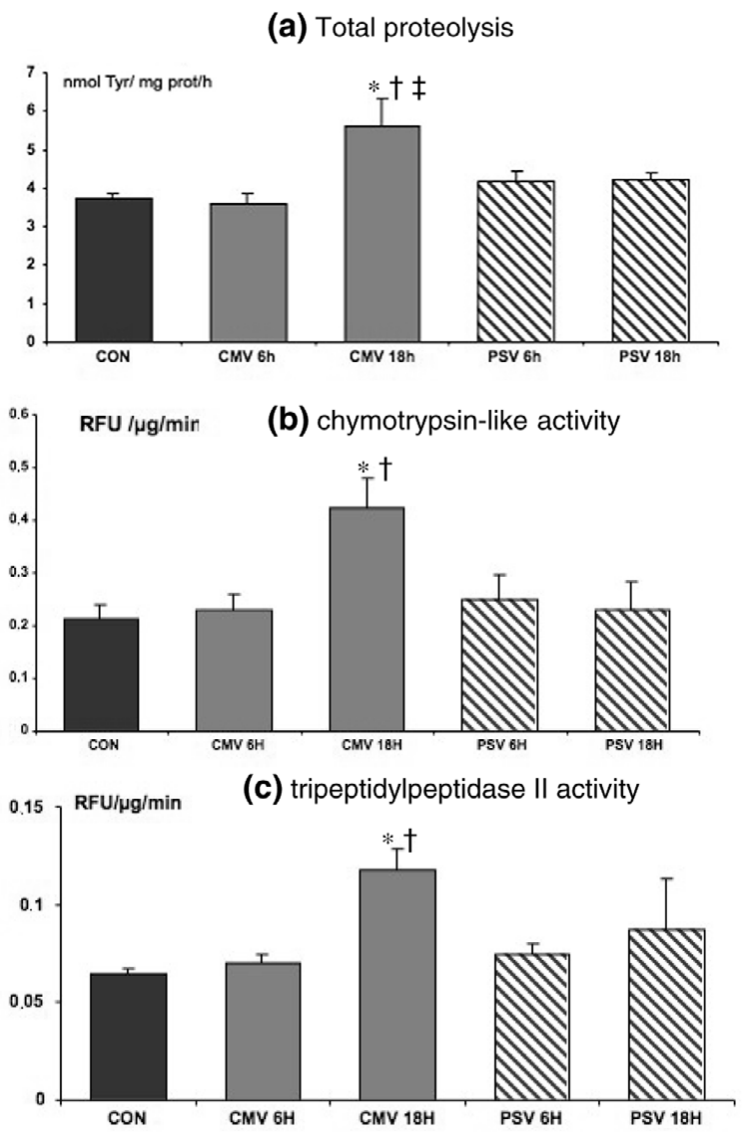
*In vitro *diaphragmatic proteolysis. **(a) **Controlled mechanical ventilation (CMV) increased total diaphragmatic proteolysis after 18 hours, but not after 6 hours, of mechanical ventilation versus control (CON) and pressure support ventilation (PSV). Units in (a) are nanomoles of tyrosine per milligram of protein per hour. Both chymotrypsin-like activity **(b) **and tripeptidylpeptidase II activity **(c) **were increased by 18 hours of CMV. Units in (b) and (c) are relative fluorescence units (RFU) per microgram per minute. Values are mean ± standard error. *P < 0.05 compared with CON group. ^†^*P *< 0.05 compared with PSV group at 6 and 18 hours. ^‡^*P *< 0.05 compared with CMV group at 6 hours.

Compared with control animals, diaphragmatic protein catabolism was significantly increased after 18 hours of CMV (33%, *P *= 0.0001) but not after 6 hours (Figure 2). There was a 36% increase in proteolysis between 6 and 18 hours of CMV (*P *= 0.0003). Compared with CMV, 6 and 18 hours of PSV showed no significant increase in proteolysis. Moreover, duration of PSV had no effect on total proteolysis evolution (4.18 ± 0.20 and 4.23 ± 0.12 nmol of tyrosine per milligram of protein per hour after 6 and 18 hours, respectively). Both chymotrypsin-like and tripeptydyl-peptidase 20S proteasome activities were increased after 18 hours of CMV (+50% versus controls and +45% versus CMV 6 hours). PSV did not increase 20S proteasome activities, regardless of the ventilation duration (6 or 18 hours).

### *In vitro *protein synthesis

**Figure 3:**
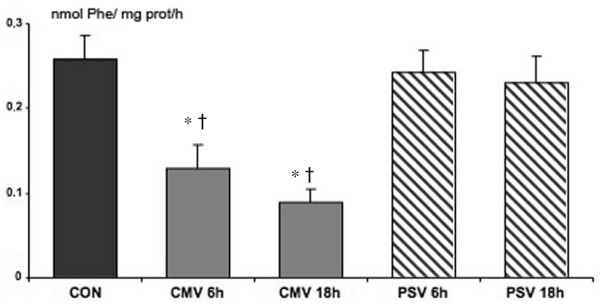
*In vitro *protein synthesis after 6 and 18 hours of controlled mechanical ventilation (CMV) and pressure support ventilation (PSV). Units are nanomoles of phenylalanine (Phe) per milligram of protein per hour. Values are mean ± standard error. *P < 0.05 compared with control (CON) group. ^†^*P *< 0.05 compared with PSV group at 6 and 18 hours.

Compared with control animals, CMV decreased diaphragmatic protein synthesis by 50% (*P *= 0.0012) after 6 hours and by 65% (*P *< 0.0001) after 18 hours of MV (Figure 3). The difference between 6 and 18 hours of CMV was 30%, which was not statistically significant. No variation of protein synthesis was observed during PSV. After 18 hours of MV, CMV showed a 94% reduction in protein synthesis compared with PSV (*P *= 0.0002).

### Measurement of diaphragm oxidative injury

**Figure 4:**
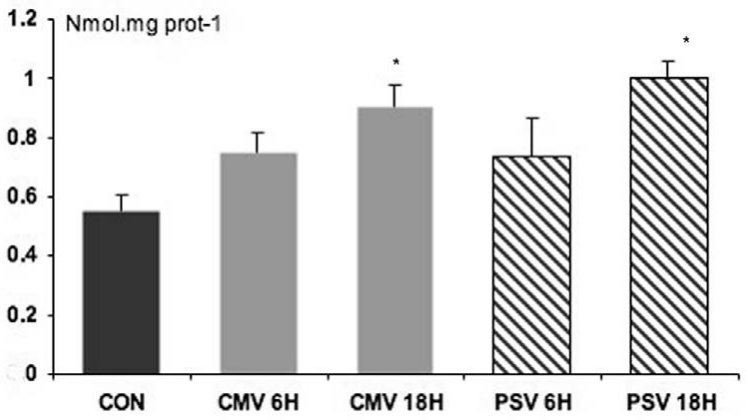
Protein-carbonyl content after 6 and 18 hours of controlled mechanical ventilation (CMV) and pressure support ventilation (PSV). Units are nanomoles per milligram of protein. Values are mean ± standard error. *P < 0.05 compared with control (CON) group.

Compared with control animals, protein oxidation, measured by myofibrillar protein carbonyl levels, was significantly increased after 18 hours of CMV (+63%, *P *< 0.001) and PSV (+82%, *P *< 0.0005) (Figure 4). Myofibrillar protein oxidation was not influenced by ventilator mode.

## Discussion

The major finding of this study, which is the first to compare PSV with control ventilation, is that, in contrast to CMV, PSV did not increase diaphragmatic muscle proteolysis or decrease protein synthesis. Both of these effects have been shown to occur as a result of CMV-induced muscle atrophy [2,11]. Finally, our results support the hypothesis that oxidative injury, though indisputable, is probably not the trigger of CMV-induced diaphragmatic proteolytic damage and thus of VIDD. Before discussion of the results, some study limitations must be pointed out.

### Anesthetic protocol

The anesthetic agent, sodium pentobarbital, could have affected the rate of muscle protein synthesis in the diaphragm. However, both MV and spontaneously breathing animals were anesthetized with sodium pentobarbital, so comparisons between groups are valid. Moreover, a previous study has reported that rats acutely anesthetized with sodium pentobarbital do not experience a significant decrease in protein synthesis in skeletal muscle [36]. Additionally, general anesthesia does not decrease protein synthesis in skeletal muscle in healthy humans undergoing abdominal surgery [37]. Collectively, these data indicate that protein synthesis is not altered by anesthesia *per se*. The influence of continued exposure of any given anesthetic agent (for example, 18 hours) would be difficult to separate from the reduced use during that state. However, the experiments reviewed above [36,37] report normal rates of protein synthesis in limb-locomotor skeletal muscle during periods of time in which reduced use would not be expected to have an effect on protein synthesis. These reports [36,37] indicate that anesthesia does not affect protein synthesis; therefore, the decreased rate of protein synthesis in the diaphragm during MV is attributable to MV, not to the anesthetic as previously reported by several authors [2,4,6,11,38].

### Diaphragmatic contraction

Prolonged MV results in diaphragmatic atrophy and contractile dysfunction in animals. Evaluation of contractile diaphragmatic properties in PSV and CMV will have been clinically relevant. This study was not designed to respond to this question and we discuss only MV-induced diaphragmatic protein alterations. Further studies should focus on this point. Diaphragmatic contractions are avoided by CMV at a normal rate (80 cycles per minute). We have not tested this assessment but several authors have done so previously [4] and used this previously reviewed paper for a recent study [2,11]. However, this does not exclude the possibility that the animals were triggering the ventilator during CMV in the present study. This is a real limitation of the manuscript.

### Kinetics of controlled mechanical ventilation-induced protein metabolism alteration

In the present study, we simultaneously analyze the effects of MV on proteolysis, protein synthesis, and their kinetics. Consistent with earlier findings [2], our results confirm the increase in diaphragmatic proteolysis after 18 hours of CMV. Although diaphragmatic proteolytic injury has been implicated in the genesis of VIDD [7], less is known about modifications in diaphragmatic protein synthesis as a result of MV. Muscle atrophy can result from increased proteolysis [39], decreased protein synthesis [40], or both. Except for one recent study [11], none had considered the possibility that diaphragm atrophy associated with CMV could also result from decreased protein synthesis. We found both increased proteolysis and a time-dependent decrease in protein synthesis. Moreover, our results provide information about the probable kinetics of CMV-induced protein metabolism modifications. Indeed, the decrease in protein synthesis occurred extremely early (by the sixth hour of CMV), was worsened by the duration of MV, and preceded the increase in diaphragmatic proteolysis. It is interesting to note that, in the study of Shanely and colleagues [11], the results were obtained from the analysis of separate studies of *in vitro *proteolysis and *in vivo *protein synthesis. However, constant infusion of ^13^C-leucine, which is used in the analysis of *in vivo *protein synthesis, can modify an animal's protein profile by altering insulin release, on both the tissue and molecular levels [41], making interpretations between *in vivo *and *in vitro *models difficult. In addition, the nutritional profiles of animals can limit the interpretation. Indeed, some authors have compared the results obtained using fed [2] and unfed animals, implying a negative protein assessment [11,41]. On the other hand, *in vivo *protein synthesis should be more relevant than *in vitro *proteolysis as used in our study. These methodological differences could explain some difference in the results.

### Pressure support ventilation-induced diaphragmatic exercise

Our data showed that PSV limits MV-induced increases in proteolysis and decreases in protein synthesis. Moreover, in contrast to CMV, modifications in protein metabolism were not affected by PSV duration. Because of differences in proteolysis/protein synthesis ratios, we hypothesized that PSV allows the maintenance of protein turnover. In addition, because CMV decreased protein synthesis, it is likely that CMV decreases or completely inhibits protein turnover. These differences in modification of metabolism may be due to differences in the type of diaphragmatic muscle damage caused by CMV and PSV. Indeed, as for peripheral skeletal muscle models, during PSV the diaphragm is subjected to exercise type activity through an increase in respiratory activity (versus CMV) [42-44]. This exercise would protect the diaphragm from modifications related to muscular inactivity caused by CMV. During CMV, there is a complete absence of neural activation and mechanical activity in the diaphragm [4,45], which undergoes passive shortening during mechanical expansion of the lungs [46,47]. This trauma has been implicated in the genesis of VIDD [2,11], in particular during sarcomere injury [48,49] and during decreased force-generating capacity of the diaphragm [7,50]. There has been little determination of the types of proteins implicated in CMV-induced metabolic damage. CMV has been shown to decrease the rate of mixed muscle protein synthesis by 30% and to decrease the rate of myosin heavy chain protein synthesis by 65% [11]. Although our study was not designed to analyze the type of proteins involved in the reduction of protein synthesis, it shed new light on the changes in protein synthesis associated with the conservation of diaphragm activity. Further experiments are necessary to determine the specific proteins implicated in the increased protein turnover observed with PSV. Our results also confirm that the 20S proteasome is involved in MV-induced proteolytic damage [2,10]. CMV increases 20S proteasome activity in parallel with the increase in diaphragmatic proteolysis. After 18 hours of CMV, we observed an increase in the activity of extralysosomal TPPII, which degrades peptides generated by the proteasome. Similarly, 72 hours of CMV increased the level of MAF-box mRNA, which encodes an E3 ligase implicated in the ubiquitination of proteins targeted for degradation via the proteasome [38]. Together, these findings indicate the importance of the ubiquitin-proteasome pathway in CMV-induced diaphragmatic muscle damage and in overall regulation of muscle proteolysis [51] (as well as the importance of this enzymatic system within the skeletal muscle proteolytic machinery [52,53]).

### Is protein oxidation a real trigger?

Little is currently known concerning the triggers or molecular signals of MV-induced protein metabolism modifications and muscle atrophy [51,54]. Oxidative injury is induced by MV, and increased protein oxidation and lipid peroxidation were found to be associated with CMV [2,55]. Oxidative stress occurs within a few hours after the start of CMV [9,56] and may play a central role in the pathogenesis of CMV-induced diaphragmatic atrophy [7]. Oxidized proteins are associated with increased proteolysis, which generates muscle atrophy and dysfunction [57,58]. Because PSV does not increase proteolysis (contrary to CMV) or decrease protein synthesis, it is likely that PSV causes less oxidative injury. Our results confirm that CMV is associated with diaphragmatic oxidative stress as indicated by an increase in protein myofibrillar oxidation. The increase in protein carbonyl levels parallels the increase in 20S proteasome activity, which specializes in degrading proteins oxidized by reactive oxygen species [7,59]. Thus, oxidized proteins may generate an increase in 20S proteasome activity. Contrary to our hypothesis, we observed a similar oxidation of myofibrillar protein with PSV. Thus, even if MV causes oxidative stress, our findings support the hypothesis that protein oxidation probably does not trigger the diaphragmatic proteolytic damage generated by CMV and its associated diaphragmatic dysfunction. Nevertheless, an overproduction of free radicals may constitute the molecular signal of CMV-increased proteolysis, either in mitochondria (as suggested by an increase in manganese-superoxide dismutase activity [9]) or via other metabolic pathways (such as that involving xanthine oxidase [12]). There is also the possibility that other diaphragmatic regulating factors (such as apoptosis) might be involved [60].

## Conclusion

We confirm that, within a few hours, CMV alters diaphragmatic muscle protein metabolism. CMV first reduces protein synthesis and then increases proteolysis. Compared with CMV, PSV limits muscle wasting through a better protein balance despite marked oxidative stress. If further study confirms our biochemical findings with histological and electromyographical data, PSV may be an alternative to CMV to limit muscle atrophy and diaphragmatic dysfunction.

## Key messages

• Controlled mechanical ventilation reduces protein synthesis and secondly increases proteolysis.

• Pressure support ventilation limits muscle wasting through a better protein balance.

• Pressure Support Ventilation may be an alternative to Controlled mechanical Ventilation to limit diaphragmatic atrophy.

## Abbreviations

^14^C-Phe: ^14^C-phenylalanine; AAF: alanine-alanine-phenylalanine; AMC: 7-amino-4-methylcoumarin; CMV: controlled mechanical ventilation; DNPH: 2,4-dinitrophenylhydrazones; DTT: dithiothreitol; FiO_2_: fraction of inspired oxygen; LLVY: leucine-leucine-valine-tyrosine; MV: mechanical ventilation; PSV: pressure support ventilation; TCA: trichloroacetic acide; TPPII: tripeptidylpeptidase II; VIDD: ventilator-induced diaphragm dysfunction.

## Competing interests

The authors declare that they have no competing interests.

## Authors' contributions

EF and J-MC participated in the design of the study, carried out the study, and helped to draft the manuscript. They contributed equally to this work. LC, LM, LR, VS, and DA participated in the design of the study, performed biochemical analysis, and helped to draft the manuscript. SJ, BJ and J-EB participated in the design of the study and helped to draft the manuscript. All authors read and approved the final manuscript.

## References

[B1] Levine S, Nguyen T, Taylor N, Friscia ME, Budak MT, Rothenberg P, Zhu J, Sachdeva R, Sonnad S, Kaiser LR, Rubinstein NA, Powers SK, Shrager JB (2008). Rapid disuse atrophy of diaphragm fibers in mechanically ventilated humans. N Engl J Med.

[B2] Shanely RA, Zergeroglu MA, Lennon SL, Sugiura T, Yimlamai T, Enns D, Belcastro A, Powers SK (2002). Mechanical ventilation-induced diaphragmatic atrophy is associated with oxidative injury and increased proteolytic activity. Am J Respir Crit Care Med.

[B3] Sassoon CS (2002). Ventilator-associated diaphragmatic dysfunction. Am J Respir Crit Care Med.

[B4] Sassoon CS, Caiozzo VJ, Manka A, Sieck GC (2002). Altered diaphragm contractile properties with controlled mechanical ventilation. J Appl Physiol.

[B5] Vassilakopoulos T, Zakynthinos S, Roussos C (2006). Bench-to-bedside review: weaning failure – should we rest the respiratory muscles with controlled mechanical ventilation?. Crit Care.

[B6] Vassilakopoulos T (2008). Ventilator-induced diaphragm dysfunction: the clinical relevance of animal models. Intensive Care Med.

[B7] Vassilakopoulos T, Petrof BJ (2004). Ventilator-induced diaphragmatic dysfunction. Am J Respir Crit Care Med.

[B8] Lemaire F (1993). Difficult weaning. Intensive Care Med.

[B9] Shanely RA, Coombes JS, Zergeroglu AM, Webb AI, Powers SK (2003). Short-duration mechanical ventilation enhances diaphragmatic fatigue resistance but impairs force production. Chest.

[B10] DeRuisseau KC, Shanely RA, Akunuri N, Hamilton MT, Van Gammeren D, Zergeroglu AM, McKenzie M, Powers SK (2005). Diaphragm unloading via controlled mechanical ventilation alters the gene expression profile. Am J Respir Crit Care Med.

[B11] Shanely RA, Van Gammeren D, Deruisseau KC, Zergeroglu AM, McKenzie MJ, Yarasheski KE, Powers SK (2004). Mechanical ventilation depresses protein synthesis in the rat diaphragm. Am J Respir Crit Care Med.

[B12] Kondo H, Nakagaki I, Sasaki S, Hori S, Itokawa Y (1993). Mechanism of oxidative stress in skeletal muscle atrophied by immobilization. Am J Physiol.

[B13] Li YP, Chen Y, Li AS, Reid MB (2003). Hydrogen peroxide stimulates ubiquitin-conjugating activity and expression of genes for specific E2 and E3 proteins in skeletal muscle myotubes. Am J Physiol Cell Physiol.

[B14] Betters JL, Criswell DS, Shanely RA, Van Gammeren D, Falk D, Deruisseau KC, Deering M, Yimlamai T, Powers SK (2004). Trolox attenuates mechanical ventilation-induced diaphragmatic dysfunction and proteolysis. Am J Respir Crit Care Med.

[B15] Maes K, Testelmans D, Powers S, Decramer M, Gayan-Ramirez G (2007). Leupeptin inhibits ventilator-induced diaphragm dysfunction in rats. Am J Respir Crit Care Med.

[B16] Gayan-Ramirez G, Testelmans D, Maes K, Racz GZ, Cadot P, Zador E, Wuytack F, Decramer M (2005). Intermittent spontaneous breathing protects the rat diaphragm from mechanical ventilation effects. Crit Care Med.

[B17] Hering R, Bolten JC, Kreyer S, Berg A, Wrigge H, Zinserling J, Putensen C (2008). Spontaneous breathing during airway pressure release ventilation in experimental lung injury: effects on hepatic blood flow. Intensive Care Med.

[B18] Jolliet P, Tassaux D (2006). Clinical review: patient-ventilator interaction in chronic obstructive pulmonary disease. Crit Care.

[B19] Brander L, Slutsky AS (2006). Assisted spontaneous breathing during early acute lung injury. Crit Care.

[B20] Conti G, Arcangeli A, Antonelli M, Cavaliere F, Costa R, Simeoni F, Proietti R (2004). Sedation with sufentanil in patients receiving pressure support ventilation has no effects on respiration: a pilot study. Can J Anaesth.

[B21] Brochard L, Pluskwa F, Lemaire F (1987). Improved efficacy of spontaneous breathing with inspiratory pressure support. Am Rev Respir Dis.

[B22] Brochard L, Harf A, Lorino H, Lemaire F (1989). Inspiratory pressure support prevents diaphragmatic fatigue during weaning from mechanical ventilation. Am Rev Respir Dis.

[B23] National Research Council (1996). Guide for the Care and Use of Laboratory Animals.

[B24] Le Bourdelles G, Viires N, Boczkowski J, Seta N, Pavlovic D, Aubier M (1994). Effects of mechanical ventilation on diaphragmatic contractile properties in rats. Am J Respir Crit Care Med.

[B25] Schnader JY, Juan G, Howell S, Fitzgerald R, Roussos C (1985). Arterial CO_2 _partial pressure affects diaphragmatic function. J Appl Physiol.

[B26] Tischler ME, Desautels M, Goldberg AL (1982). Does leucine, leucyl-tRNA, or some metabolite of leucine regulate protein synthesis and degradation in skeletal and cardiac muscle?. J Biol Chem.

[B27] Waalkes TP, Udenfriend S (1957). A fluorometric method for the estimation of tyrosine in plasma and tissues. J Lab Clin Med.

[B28] Temparis S, Asensi M, Taillandier D, Aurousseau E, Larbaud D, Obled A, Bechet D, Ferrara M, Estrela JM, Attaix D (1994). Increased ATP-ubiquitin-dependent proteolysis in skeletal muscles of tumor-bearing rats. Cancer Res.

[B29] Smith PK, Krohn RI, Hermanson GT, Mallia AK, Gartner FH, Provenzano MD, Fujimoto EK, Goeke NM, Olson BJ, Klenk DC (1985). Measurement of protein using bicinchoninic acid. Anal Biochem.

[B30] Combaret L, Tilignac T, Claustre A, Voisin L, Taillandier D, Obled C, Tanaka K, Attaix D (2002). Torbafylline (HWA 448) inhibits enhanced skeletal muscle ubiquitin-proteasome-dependent proteolysis in cancer and septic rats. Biochem J.

[B31] Hobler SC, Williams A, Fischer D, Wang JJ, Sun X, Fischer JE, Monaco JJ, Hasselgren PO (1999). Activity and expression of the 20S proteasome are increased in skeletal muscle during sepsis. Am J Physiol.

[B32] Wray CJ, Tomkinson B, Robb BW, Hasselgren PO (2002). Tripeptidyl-peptidase II expression and activity are increased in skeletal muscle during sepsis. Biochem Biophys Res Commun.

[B33] Fang CH, Li BG, Fischer DR, Wang JJ, Runnels HA, Monaco JJ, Hasselgren PO (2000). Burn injury upregulates the activity and gene expression of the 20 S proteasome in rat skeletal muscle. Clin Sci (Lond).

[B34] Lowry OH, Rosebrough NJ, Farr AL, Randall RJ (1951). Protein measurement with the Folin phenol reagent. J Biol Chem.

[B35] Fagan JM, Sleczka BG, Sohar I (1999). Quantitation of oxidative damage to tissue proteins. Int J Biochem Cell Biol.

[B36] Heys SD, Norton AC, Dundas CR, Eremin O, Ferguson K, Garlick PJ (1989). Anaesthetic agents and their effect on tissue protein synthesis in the rat. Clin Sci (Lond).

[B37] Essen P, McNurlan MA, Wernerman J, Vinnars E, Garlick PJ (1992). Uncomplicated surgery, but not general anesthesia, decreases muscle protein synthesis. Am J Physiol.

[B38] Sassoon CS, Zhu E, Caiozzo VJ (2004). Assist-control mechanical ventilation attenuates ventilator-induced diaphragmatic dysfunction. Am J Respir Crit Care Med.

[B39] Bodine SC, Latres E, Baumhueter S, Lai VK, Nunez L, Clarke BA, Poueymirou WT, Panaro FJ, Na E, Dharmarajan K, Pan ZQ, Valenzuela DM, DeChiara TM, Stitt TN, Yancopoulos GD, Glass DJ (2001). Identification of ubiquitin ligases required for skeletal muscle atrophy. Science.

[B40] Ku Z, Yang J, Menon V, Thomason DB (1995). Decreased polysomal HSP-70 may slow polypeptide elongation during skeletal muscle atrophy. Am J Physiol.

[B41] Beaufrère B (2002). [Amino acid metabolism in normal individuals]. Journ Annu Diabetol Hotel Dieu.

[B42] Ji LL, Stratman FW, Lardy HA (1988). Enzymatic down regulation with exercise in rat skeletal muscle. Arch Biochem Biophys.

[B43] Wakshlag JJ, Kallfelz FA, Barr SC, Ordway G, Haley NJ, Flaherty CE, Kelley RL, Altom EK, Lepine AJ, Davenport GM (2002). Effects of exercise on canine skeletal muscle proteolysis: an investigation of the ubiquitin-proteasome pathway and other metabolic markers. Vet Ther.

[B44] Stupka N, Tarnopolsky MA, Yardley NJ, Phillips SM (2001). Cellular adaptation to repeated eccentric exercise-induced muscle damage. J Appl Physiol.

[B45] Powers SK, Shanely RA (2002). Exercise-induced changes in diaphragmatic bioenergetic and antioxidant capacity. Exerc Sport Sci Rev.

[B46] Froese AB, Bryan AC (1974). Effects of anesthesia and paralysis on diaphragmatic mechanics in man. Anesthesiology.

[B47] Newman S, Road J, Bellemare F, Clozel JP, Lavigne CM, Grassino A (1984). Respiratory muscle length measured by sonomicrometry. J Appl Physiol.

[B48] Williams PE, Goldspink G (1976). The effect of denervation and dystrophy on the adaptation of sarcomere number to the functional length of the muscle in young and adult mice. J Anat.

[B49] Farkas GA, Roussos C (1983). Diaphragm in emphysematous hamsters: sarcomere adaptability. J Appl Physiol.

[B50] Yang L, Luo J, Bourdon J, Lin MC, Gottfried SB, Petrof BJ (2002). Controlled mechanical ventilation leads to remodeling of the rat diaphragm. Am J Respir Crit Care Med.

[B51] Attaix D, Combaret L, Pouch MN, Taillandier D (2001). Regulation of proteolysis. Curr Opin Clin Nutr Metab Care.

[B52] Attaix D, Combaret L, Kee AJ, Taillandier D, Zempleni J, Daniel H (2003). Mechanisms of ubiquitination and proteasome-dependent proteolysis in skeletal muscle. Molecular Nutrition.

[B53] Taillandier D, Combaret L, Pouch MN, Samuels SE, Bechet D, Attaix D (2004). The role of ubiquitin-proteasome-dependent proteolysis in the remodelling of skeletal muscle. Proc Nutr Soc.

[B54] Jackman RW, Kandarian SC (2004). The molecular basis of skeletal muscle atrophy. Am J Physiol Cell Physiol.

[B55] Jaber S, Sebbane M, Koechlin C, Hayot M, Capdevila X, Eledjam JJ, Prefaut C, Ramonatxo M, Matecki S (2005). Effects of short vs. prolonged mechanical ventilation on antioxidant systems in piglet diaphragm. Intensive Care Med.

[B56] Zergeroglu MA, McKenzie MJ, Shanely RA, Van Gammeren D, DeRuisseau KC, Powers SK (2003). Mechanical ventilation-induced oxidative stress in the diaphragm. J Appl Physiol.

[B57] Nagasawa T, Hatayama T, Watanabe Y, Tanaka M, Niisato Y, Kitts DD (1997). Free radical-mediated effects on skeletal muscle protein in rats treated with Fe-nitrilotriacetate. Biochem Biophys Res Commun.

[B58] Dean RT, Fu S, Stocker R, Davies MJ (1997). Biochemistry and pathology of radical-mediated protein oxidation. Biochem J.

[B59] Hussain SN, Vassilakopoulos T (2002). Ventilator-induced cachexia. Am J Respir Crit Care Med.

[B60] McClung JM, Kavazis AN, DeRuisseau KC, Falk DJ, Deering MA, Lee Y, Sugiura T, Powers SK (2007). Caspase-3 regulation of diaphragm myonuclear domain during mechanical ventilation-induced atrophy. Am J Respir Crit Care Med.

